# Curriculum Mapping to Enhance Antiracism Education in an Undergraduate Medical Education Program

**DOI:** 10.7759/cureus.62089

**Published:** 2024-06-10

**Authors:** Grace Qiu, Dimitrios Papanagnou, Bernard Lopez

**Affiliations:** 1 Medicine, Thomas Jefferson University, Philadelphia, USA; 2 Emergency Medicine, Thomas Jefferson University, Philadelphia, USA

**Keywords:** curriculum development and evaluation, diversity and inclusion, undergraduate medical education, antiracism, curriculum mapping

## Abstract

Antiracism education (ARE) is critical in developing culturally competent physicians. At our institution, the Sidney Kimmel Medical College (SKMC) at Thomas Jefferson University in Philadelphia, United States, the Office of Diversity and Inclusion Initiatives and Educational Leadership created and examined a map of its ARE curriculum. Our efforts were meant to describe our local educational processes with regards to ARE; we did not intend to compare our curriculum and its outputs to national benchmarks. To this effect, diversity deans of other local Philadelphia-area medical schools were queried on their respective ARE maps and educational offerings. Potential changes to SKMC's ARE would be considered, but no other school that was queried had a formal ARE map in place. While all schools had a variety of lectures, modules, and electives, none appeared to have a systematic method to teach ARE. As a result, modifications to SKMC's ARE were made based on an intrinsic examination of its own ARE map. Changes that were made included modifying a pre-clerkship lecture on "Racism and Microaggressions" to a small group discussion session. Additionally, a clerkship-specific lecture on "Bias and Microaggressions" was changed from four 1-hour lectures to 90 minutes of lecture followed by a 2-hour small group session, to reduce content redundancy and promote more student reflection. For both of these changes, faculty participated in a newly developed faculty development session. To guide prospective work, a multidisciplinary task force was created to include formal student input in the process of examining ARE. Future directions to query institutions outside the Philadelphia region for their ARE offerings will also be considered.

## Editorial

Teaching antiracism in medical education is fundamental to training culturally competent healthcare professionals. The authors, who represent medical student and faculty educator stakeholders, aimed to identify areas within the curriculum that require improvement at our institution, the Sidney Kimmel Medical College (SKMC) at Thomas Jefferson University in Philadelphia, United States. Our rationale was to create a map of antiracism education (ARE) across the entire curriculum to allow us to examine the breadth and depth of our ARE initiatives in our undergraduate medical education program and allow us to determine potential areas for improvement and change.

Studies have found that there have been both student-driven and faculty-driven initiatives to help design and implement these curricular revisions for ARE training [1-3]. For example, student-driven initiatives have included collaboration with faculty to develop a microaggressions workshop for case-based learning, and emphasizing how to respond to future encounters to improve the students’ learning environment [1,4]. Examples of faculty-driven initiatives have included structural racism pilot curricula as a means to better understand implementation challenges and improve ARE [3,5]. These approaches rely heavily on collaboration between faculty and students to successfully identify and address educational gaps for improvement [1-3].

To try and identify the gaps in ARE at our institution, SKMC's Office of Diversity and Inclusion Initiatives, in conjunction with the medical school's leadership, first mapped out the medical school curriculum (JeffMD) to identify individual components of ARE that already exist across the JeffMD curriculum. Our initial interest was to compare antiracism curricula between JeffMD and those of other medical schools in the region. Informal surveys to the four allopathic medical schools in the greater Philadelphia were distributed, which collected information on how these schools organize and structure their antiracism curricula. The goal was to potentially collaborate with other institutions within the region to identify gaps within ARE to help identify potential opportunities for curricular revision and improvement.

The JeffMD curriculum is organized into three phases such as foundational basic and clinical sciences during the first two years of training (phase 1); core clinical clerkship rotations during the third year of training (phase 2); and advanced clinical clerkships and career exploration and preparation during the fourth year of training (phase 3). While each phase focuses on a different aspect of students' medical training, ARE is embedded vertically and longitudinally across all three phases through the school's Health Systems Science (HSS) thread. During phase 1, topics of unconscious bias, microaggressions, structural bias in medicine, and the effects of bias and racism on the social determinants of health are taught through lectures, case-based learning sessions, small group discussions, as well as simulations. During phase 2, similar topics are taught through clerkship-specific modules, and a full-day, all-class program when students change clerkships (“interclerkship session”). Last, phase 3 includes additional lectures and interactive sessions to connect topics covered during phases 1 and 2 to students' clinical practice to prepare them for their graduate training in residency.

The creation of the ARE map has allowed for better coordination of ARE education across the JeffMD curriculum and has allowed us to better ensure the use of educational methods relevant to our students' development. We were able to use the ARE map to successfully make changes at each phase of the curriculum. For example, the following phase-specific modifications were made to our curriculum based on this mapping: the phase 1 lecture on “Racism and Microaggressions” was modified to a lecture followed by a case-based learning (CBL) session; the phase 2 interclerkship session on “Bias and Microaggressions” was changed from four 1-hour lectures to a 90-minute lecture followed by a 2-hour small group CBL session. The purpose of these curricular modifications was to not only remove redundancy in lecture content but also provide more opportunities for student reflection and discussion in smaller group settings. For both of these modifications, faculty participated in a newly developed faculty development session to prepare them for group conversations and facilitation. No other immediate changes were made to ARE. Interestingly, our survey of other regional medical schools revealed that, while each featured a variety of lectures, modules, and electives to help teach ARE, none had organized their respective ARE curriculum into a formal map.

Our office's leadership created a multidisciplinary task force of students and faculty across the institution to continuously examine current ARE offerings within the JeffMD curriculum and make ongoing recommendations about curriculum modifications as needed in real time. For example, student leadership groups were invited to meet with senior medical school leadership to discuss gaps in the curriculum on an ongoing basis. This exposed the need to better equip our faculty with the skills to successfully facilitate challenging conversations pertaining to racism and microaggressions. As a result, our offices have now developed and offer faculty development sessions in conjunction with our student-focused ARE training sessions. Future directions to query institutions outside the Philadelphia area for their respective ARE curricular offerings to compare and identify gaps within JeffMD’s curriculum are also being considered.

Even though there is no gold standard method to teach ARE, it is important to highlight that antiracism competencies and education should be viewed as an ongoing process. We call upon medical education leaders to also examine their antiracism curricular offerings and identify areas of possible revision that would enhance the quality of ARE for clinicians in training. We hope our efforts to organize and revise our ARE curriculum will better prepare trainees to effectively respond to situations and engage with individuals of different racial identities in an empathetic and culturally competent manner.
